# Supplementary material for: Detection of Hepatitis C Virus Infection from Patient Sera in Cell Culture Using Semi-Automated Image Analysis
Source: Viruses. 2024 Nov 30;16(12):1871. doi: 10.3390/v16121871 (PMC11680372; doi:10.3390/v16121871)
Supplement: Supplementary file 1 [file viruses-16-01871-s001.zip › viruses-3299921-supplementary.pdf]

Table S1. Sera used for RT-qPCR analysis only.

Serum	Date and cohort	Titer[1]	Gt	HCV RNA pi[2]
4468	2016 Frankfurt	$1{,}02 \times 10^{7}$	1b	Yes
5342	2016 Frankfurt	$5{,}78 \times 10^{5}$	1b	No
3617	2015 Frankfurt	$3{,}81 \times 10^{6}$	1b	Cells died
2054	2015 Frankfurt	$4{,}10 \times 10^{5}$	1b	No
4695	2016 Frankfurt	$2{,}97 \times 10^{5}$	1b	No
2823	2021 Frankfurt	$9{,}73 \times 10^{5}$	1b	No
3260	2015 Frankfurt	$1{,}95 \times 10^{6}$	1b	No
7437	2016 Frankfurt	$3{,}07 \times 10^{5}$	1b	No
5120	2016 Frankfurt	$7{,}64 \times 10^{5}$	1b	No
4153	2016 Frankfurt	$7{,}61 \times 10^{5}$	1b	No
4886	2016 Frankfurt	$4{,}98 \times 10^{5}$	1b	No
3676	2015 Frankfurt	$2{,}00 \times 10^{6}$	1b	No
6936	2017 Frankfurt	$6{,}52 \times 10^{5}$	1b	No
6087	2017 Frankfurt	$2{,}90 \times 10^{5}$	1b	No
4202	2016 Frankfurt	$1{,}72 \times 10^{4}$	1b	No
5613	2017 Frankfurt	$1{,}89 \times 10^{5}$	1b	No
1199	2014 Frankfurt	$7{,}24 \times 10^{3}$	1b	No
1597	2015 Frankfurt	$3{,}01 \times 10^{6}$	1b	Cells died
521	2020 Heidelberg	$7{,}85 \times 10^{5}$	1a	No
566	2020 Heidelberg	Not detectable	1b	No
574	2020 Heidelberg	$2{,}72 \times 10^{6}$	1b	No
572	2020 Heidelberg	$4{,}24 \times 10^{5}$	1b	No
77	2013 Heidelberg	$3{,}92 \times 10^{3}$	1b	No
570	2020 Heidelberg	$2{,}98 \times 10^{5}$	1b	No
579	2020 Heidelberg	$8{,}96 \times 10^{3}$	1a	No
210	2014 Frankfurt	$1{,}78 \times 10^{6}$	1b	No
GLT1	2014 Heidelberg	$4{,}22 \times 10^{7}$	1b	Yes

[1][RNA copies/mL]
[2]HCV RNA detected post infection (pi) with a Ct<36
